# Commissioning of a three‐dimensional arc‐based technique for total body irradiation

**DOI:** 10.1002/acm2.13355

**Published:** 2021-07-14

**Authors:** León G. Aldrovandi, Rubén O. Farías, María F. Mauri, María L. Mairal

**Affiliations:** ^1^ Departamento de Física Médica Mevaterapia Oncología Radiante Ciudad Autónoma de Buenos Aires Argentina

**Keywords:** 3D, Arc‐based, TBI, technique

## Abstract

The purpose of this study is to describe the commissioning of a novel three‐dimensional arc‐based technique for total body irradiation (TBI) treatments. The development and implementation of this technique allowed our institution to transition from a bilateral two‐dimensional (2D) technique to a methodology based on volumetric dose calculation. The methodology described in this work is a derivation from the MATBI technique, with the static fields being replaced by four contiguous arc‐fields for each anterior and posterior incidence. The reduced number of fields we employed makes it possible to reach a satisfactory dose uniformity through manual optimization in a straightforward process. We use the Eclipse anisotropic analytical algorithm (AAA) algorithm, commissioned with preconfigured beam data for a 6 MV photon beam, at standard SSD (100 cm). A thorough evaluation of the accuracy of the AAA algorithm at an extended distance (approximately 200 cm) was carried out. For the evaluation, we compared measured and calculated percentage depth–dose and profiles that included open‐field, penumbra, and out‐of‐field regions. The analysis was performed for both static and arc fields, taking into consideration unshielded fields and also in the presence of lung shielding blocks. End‐to‐end tests were carried out for our institutional template plan by two means: with a 2D ion chamber array detector in solid phantom and using Gafchromic films in an anthropomorphic phantom. The results obtained in this work demonstrate that the Eclipse AAA algorithm commissioned for standard treatments can be safely used with our TBI planning technique. Moreover, this technique proved to be a highly efficient path to replace conventional treatment techniques, providing a homogeneous dose distribution, dosimetric robustness, and shorter treatment times. In addition, as inherited from the MATBI technique, our methodology can be implemented in small treatment rooms, with no need for ancillary equipment.

## 1 INTRODUCTION

Total body irradiation (TBI) is a particular radiotherapy treatment where the whole body of the patient needs to be irradiated. It is a technique frequently used as part of the conditioning regimen for patients with acute myeloid leukemia or acute lymphoid leukemia undergoing hematopoietic stem cell transplantation. When TBI is to be combined with myeloablative conditioning regimens, the most common TBI schedules include twice‐daily 2‐Gy fractions given over 3 days (total dose 12 Gy); twice‐daily 1.5‐Gy fractions over 4–4.5 days (total dose 12–13.5 Gy); three‐times‐daily 1.2‐Gy fractions over 4 days (total dose 12–13.2 Gy); and once‐daily 3‐Gy fractions for 4 days (total dose 12 Gy). Moreover, patients who cannot tolerate myeloablative conditioning regimens are offered reduced‐intensity regimens consisting of 2–4 Gy given in one or two fractions.^1^, ^2^

Considering that the target volume is too large to be irradiated with conventional fields, different techniques have been developed to overcome the related constraints, where patient positioning, energy choice, dose rate, source‐to‐skin distance, and treatment field size are the main differences between them.^3^ The following recommendations to the medical physicist are common to most publications: dose uniformity in the target (entire body) should be within ±10%, any relevant dose overdosages or underdosages greater than ±5% should be recorded, and the maximum and minimum values should be well known.^1^, ^3^, ^4^, ^5^ Therefore, everything that improves dose distribution knowledge should be promoted. Because TBI is a complex radiotherapy treatment, most institutions use very simple treatment planning approaches and a homogenous dose calculation, being traditionally delivered using two‐dimensional (2D) techniques with a conventional linac (linear accelerator) using an anterior–posterior (AP/PA) or parallel‐opposed lateral beam arrangements at an extended source‐to‐surface distance (SSD).^3^ In the literature, a growing but still low number of papers deal with three‐dimensional treatment planning issues.^6^, ^7^, ^8^, ^9^, ^10^, ^11^, ^12^, ^13^

The present work describes the implementation in our institution of a TBI treatment procedure based on volumetric treatment planning using patient's specific full‐body CT images and a commercial treatment planning system (Varian Eclipse v13.6). The new methodology has as its primary aims to be able to optimize the actual dose distribution, including regions where large heterogeneities can be found (such as thorax), and to verify the achieved dose uniformity in the entire volume of the body. The technique employed in our institution has its roots in the MATBI technique, developed by the Department of Radiation Oncology, University of California San Francisco.^7^, ^8^ In the MATBI approach, the patient is alternated between supine and prone setups on a treatment couch near the floor at about 2 m SSD. Multiple static fields with a 5° gantry angle variation are positioned in an arc formation to treat the full length of the patient. The weights of the resulting beams (between 16 and 28 fields per side, depending on the patient size) are automatically optimized using the PINNACLE3 treatment planning system (TPS) with a single constraint of uniform dose to the body contour. In the case of the Eclipse TPS, it is not possible to perform a beam‐weight optimization when the anisotropic analytical algorithm (AAA) algorithm is used for volumetric dose calculation. Since a manual beam‐weight optimization of such a large number of static fields could be excessively time‐consuming, we opted for an alternative approach consisting of the use of four contiguous arcs for each AP and PA treatment. The reduced number of fields makes it possible to reach satisfactory dose uniformity through manual optimization in a straightforward process.

To our knowledge, this is the first publication presenting the aforementioned contiguous‐arc arrangement for TBI irradiation, along with the validation of the corresponding Eclipse volumetric dose calculation for this technique. We aim to demonstrate our technique and to show that the accurate delivery of homogeneous dose distribution is possible using a standard linac and commercially available technology.

## 2 MATERIALS AND METHODS

### 2.1 Arc‐based technique

#### 2.1.1 Simulation, treatment planning, and delivery

In our institution, TBI treatments are delivered with 6 MV x rays on two machines, a Varian Trilogy and a Varian 2100CD. At the time of commissioning the treatment planning system (Varian Eclipse v13.6) for standard treatments, the two linacs were accurately modeled in Eclipse using the preconfigured data provided by Varian. Despite not being purposely matched to be dosimetrically equivalent, both machine models agree within 1% in output factors and within 1%/1 mm in profile and PDD curves. This allows us to fulfill the requirement of a back‐up machine that guarantees the completion of treatments in case of machine breakdown.

For treatment planning, a full‐body CT image is acquired with the patient in the supine position. Due to the limited scan length of the CT scanner, two scans are required. The first scan, in head‐first position, covers the superior part of the patient, usually up to the knees. The second scan, in feet‐first position, acquires the rest of the patient with an overlap of around 10 cm. A setup point located close to the umbilicus is tattooed during CT simulation. This point will be used for field isocenter localization during both planning and treatment.

Once imported into Eclipse, both CT scans are registered by the overlapping region. An extended CT that includes the whole patient is created by copying the body contour from the second scan into the first one with an overwritten density of 1 g/cm^3^. It is worth noting that the regions with density override are limited to the inferior part of the body (generally, from knees to feet), which are not covered by the first scan. Lungs are automatically contoured in the extended CT image and, then, isotropically contracted 1 cm to generate an auxiliary “shield protection” structure used for the fabrication of the lung blocks.

**FIGURE 1:**
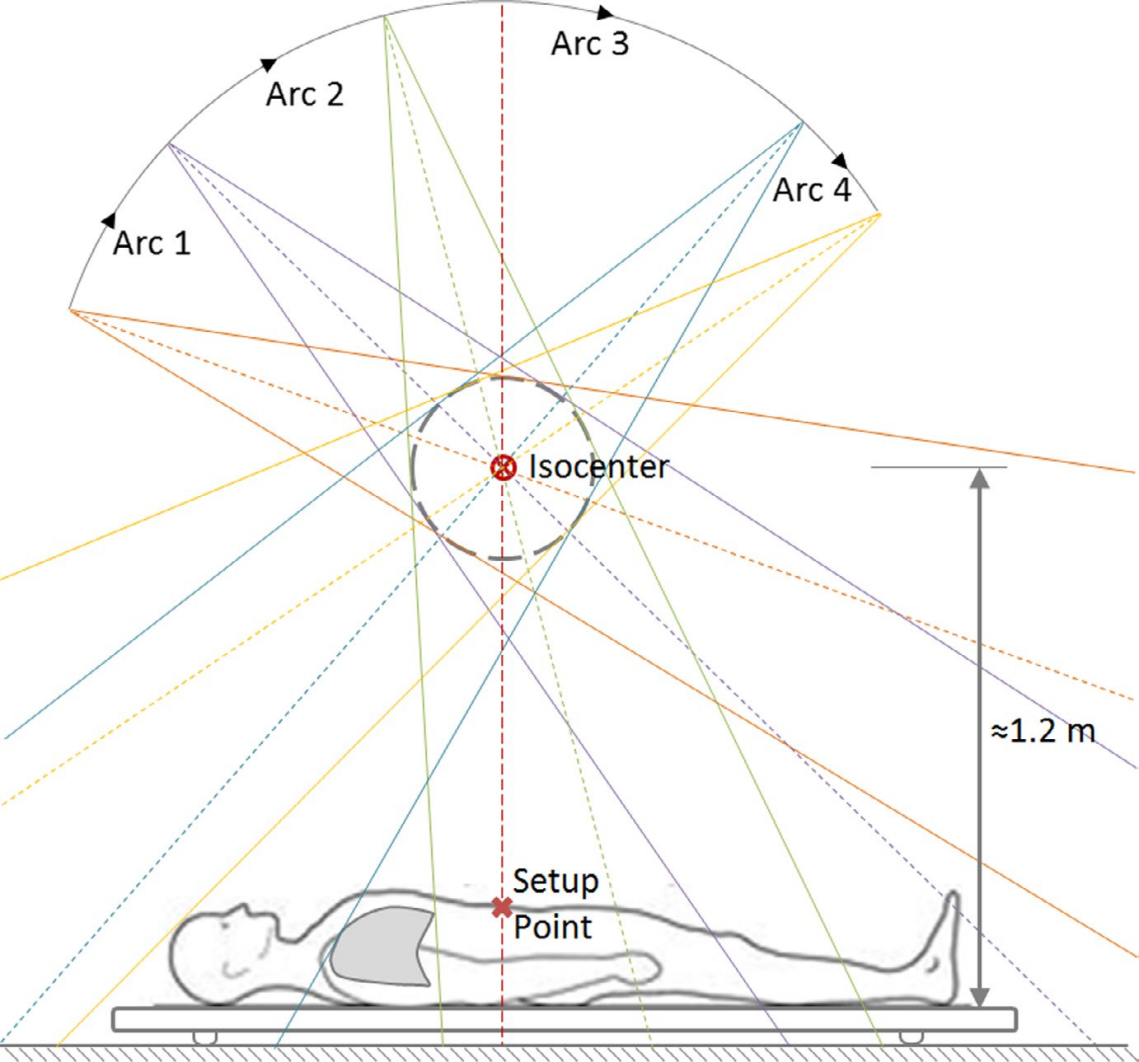
Patient position and arc‐field amplitudes for the anterior plan. Note that lungs are only irradiated by arc 3, at the lowest available dose rate (80 MU/min)

As mentioned earlier, the technique implemented in our institution has its roots in the MATBI technique, developed by the Department of Radiation Oncology, University of California San Francisco.^7^, ^8^ In our case, both anterior and posterior treatment plans consist of four contiguous arcs (Figure 1). Field size is fixed to 40 × 40 cm^2^ at the isocenter, which equates to roughly 80 × 80 cm^2^ on the couch. For all fields, lateral and longitudinal coordinates of the isocenter coincide with those of the setup point. Since treatment couch height is fixed, the isocenter‐couch distance remains unchanged for all patients (approximately 120 cm in our case). Therefore, in Eclipse, the vertical position of the isocenter is defined so that the isocenter‐couch distance matches the real distance. Angular amplitudes for arcs of each plan are chosen according to the following rule (see Figure 1):

− Arc 1: from outside the patient to the knees
− Arc 2: from the knees to the inferior border of the lungs
− Arc 3: from the inferior to the superior border of the lungs
− Arc 4: from the superior border of the lungs to outside the patient

Since lungs are only irradiated by the third arc, the lowest available dose rate (80 MU/min) was selected for this field in order to reduce the risk of pneumonitis. Moreover, a dose rate of 400 MU/min was set for the rest of the arc fields to reduce treatment delivery time. The AAA algorithm with a spatial grid of 2.5 mm and an angular resolution of 1° is used for volumetric dose calculation. Weight values for each arc can be found through the manual optimization of dose uniformity in a straightforward process. We found through a trial‐and‐error method that the configuration of four contiguous arcs defined above contains the minimum number of fields allowing satisfactory dose uniformity for a wide range of body sizes. It is worth mentioning that no modification was needed in the Eclipse AAA model commissioned at standard SSD to reach accurate dose distribution calculations at extended SSD distances, in accordance with the previous publications.^14^

For treatment delivery, the patient is positioned in a custom‐made static couch on the floor in supine and prone positions to deliver AP and PA treatments, respectively. The mid‐sagittal plane of the patient is set to coincide with the gantry rotation plane by means of treatment‐room lasers. Moreover, the longitudinal position of the patient is determined by the alignment of the center of a 0 gantry beam with the setup point defined during CT simulation. Then, lung blocks are suspended above the patient in their planned position by means of a wooden truss bridge. Before every fraction, proper block alignment is verified through a digital radiography cassette located beneath the couch. No beam spoiler is used during treatment.

#### 2.1.2 Arc‐based technique vs. MATBI and conventional AP/PA techniques

In order to evaluate how this technique compares with the MATBI and conventional AP/PA techniques, the same patient was successively planned with the three methods. In the case of the MATBI plan, field weights were derived through manual optimization instead of automatically as in the original methodology due to the aforementioned Eclipse limitations. Concerning the conventional technique, planning was performed by two AP/PA parallel opposed fields with 40 × 40 cm^2^ field size, 45° collimator angle, and an extended SSD of 370 cm so that the entire patient can be covered by the radiation field. Although the accuracy of our Eclipse model was not validated at such large SSD distances, we opted to use it to obtain the dose distribution to have a better estimation of the dose delivered to patients with this technique.

Despite the fact that lung shielding is not routinely taken into consideration during the treatment planning process of real patients, we modeled the Cerrobend blocks in this patient for every technique to be able to examine possible changes in penumbra and attenuation. In our institution, 1‐cm‐thick Cerrobend shielding is used during treatment so that the mean lung dose remains below 10 Gy (see Section 3.4.4). This shielding thickness was used for all three techniques during treatment planning. Since the isocenter position is different in each technique, lung protection was conveniently repositioned for every technique to reproduce the position it would have during respective treatments.

Dose distributions obtained for the three techniques were compared in terms of planning time, dose uniformity, mean lung dose, effective dose rate, and delivery time.

#### 2.1.3 Depth–dose curve, floor backscatter, and skin dose ‐ Static fields

For the measurement of the percentage depth–dose curve (PDD) corresponding to a 40 cm × 40 cm static field at gantry angle 0°, we used RW3 slab phantom (PTW‐Freiburg, Freiburg, Germany) with a total thickness of 25 cm at a source‐to‐surface distance (SSD) of 197.5 cm. Depths ranging from 0 to 1.5 cm were measured with a Markus plane‐parallel chamber (PTW‐Freiburg, Freiburg, Germany). In order to have a more precise determination of skin dose, over‐response of Markus chamber close to the surface was corrected according to published recommendations.^15^, ^16^ Moreover, measurements for depths varying from 1.5 to 24.2 cm were performed with an SNC600c cylindrical chamber of 0.6 cc (Sun Nuclear, Florida, USA). The use of a Farmer chamber at higher depths allowed us to have a more reliable estimation of the contribution of floor backscatter close to the couch. Once measured the depth–dose curve, the total skin dose for the full AP‐PA treatments was estimated through the addition of the contributions of both entrance and exit dose. Concerning Eclipse dose calculation accuracy of PDD for 40 cm × 40 cm static field at an extended distance, this was evaluated by means of one‐dimensional gamma index analysis.

#### 2.1.4 Beam quality index and reference absolute output

The reference conditions chosen for the determination of the reference absorbed dose to water at an extended distance were: field size 40 cm × 40 cm, depth 10 cm, and SSD 197.5 cm. Measurements were performed with an SNC600c cylindrical chamber, calibrated in the national Secondary Standard Dosimetry Laboratory (SSDL). For the measurement of the beam quality index TPR_20,10_, necessary to determine the beam quality correction factor,^17^ we used a source‐to‐detector distance (SDD) of 207.5 cm and a field size of 4.8 cm × 4.8 cm at isocenter, which gives a 10 cm × 10 cm field size at that SDD.

#### 2.1.5 Beam profile, penumbra, and out‐of‐field dose

**FIGURE 2:**
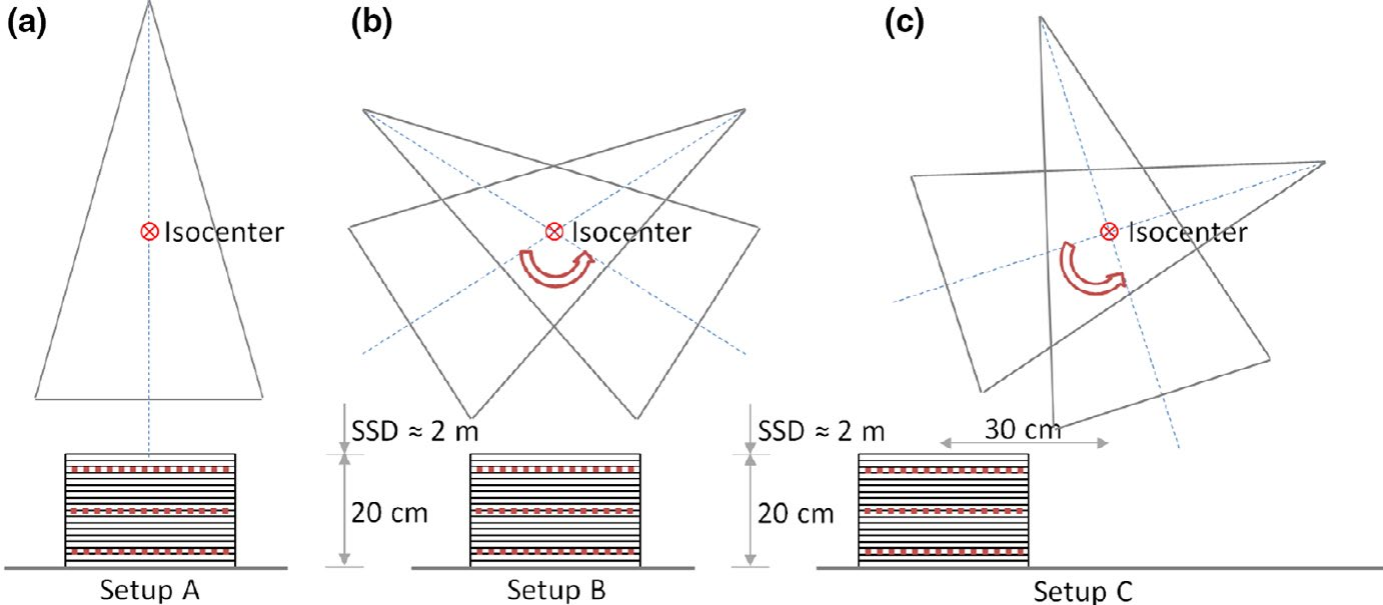
Setup used to measure beam profiles, penumbra, and out‐of‐field dose for three situations of increasing complexity: (a) static field, (b) arc field, and (c) arc field with the phantom 30 cm away from the 0 gantry beam axis. Each detector seven29 position is shown in red dash line

In order to evaluate Eclipse dose calculation accuracy outside the beam axis, including penumbra and out‐of‐field regions, we used a 2D ion chamber array detector seven29 (PTW‐Freiburg, Freiburg, Germany) embedded in RW3 slab phantom emulating a patient thickness of 20 cm. Planar dose measurements were performed at depths of 2.5, 10, and 17.5 cm at an SSD of 202.5 cm. The field size was set to 8 cm ×40 cm at isocenter (approximately 16 cm ×80 cm at detector position) so as to simultaneously measure beam profiles, penumbra, and out‐of‐field dose. This analysis was repeated in three cases of increasing complexity (Figure 2a–c): static field (setup A), arc field (setup B), and arc field with phantom positioned 30 cm away from the 0 gantry beam axis in the longitudinal direction (setup C). The displacement in the third setup was chosen to be 30 cm because this is the distance we typically find in TBI patients between the setup point and the lung mid‐height.

#### 2.1.6 Depth–dose curve for arc fields

Dose calculation accuracy of depth–dose for arc fields could be compromised by, among other causes, incorrect modeling of off‐axis softening and/or out‐of‐field dose contribution, the intrinsic complexity of calculation at an extended distance, and the angular discretization made by the TPS. From the previous measurements with the 2D detector array seven29, we analyzed Eclipse accuracy in the three situations mentioned above (setup A, B, and C).

### 2.2 Evaluation of dose calculation for the complete treatment plan

The next step was to evaluate dose calculation accuracy for a typical treatment plan, generated from the template routinely used in patients. This was implemented by two different means. On the one hand, an array detector seven29 embedded in the RW3 slab phantom was employed to measure dose distribution in the mid‐coronal plane. On the other hand, we used an Alderson Radiation Therapy (ART) anthropomorphic phantom (Radiology Support Devices Inc., California, USA) with EBT3 Gafchromic films (Ashland Advanced Materials, New Jersey, USA) inserted at different heights to measure axial dose planes.

#### 2.2.1 Coronal planar analysis in solid phantom

**FIGURE 3:**
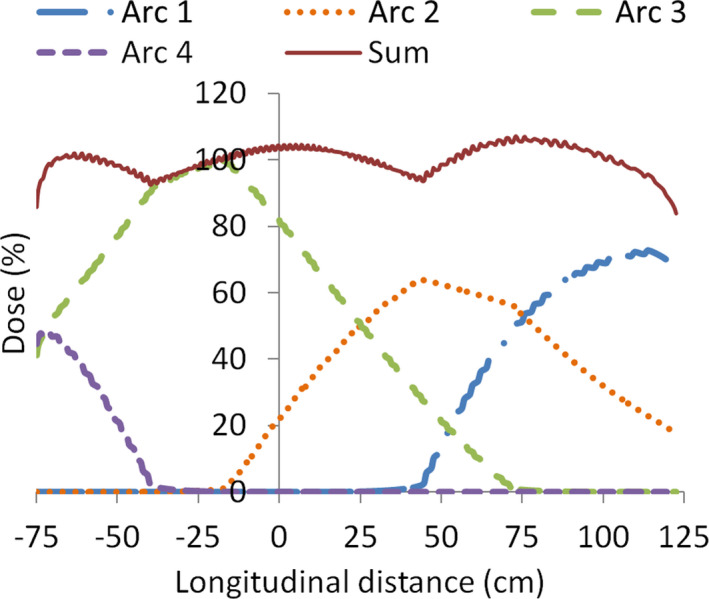
Percentage dose profiles corresponding to the longitudinal central axis in a virtual phantom. It can be noticed the dose contribution of every arc, in particular how the lung region (dashed area in the figure) is irradiated only by arc 3

A typical treatment plan was calculated on a box‐shaped virtual phantom created in Eclipse with the electron density of water and dimensions of 200‐cm length, 40‐cm width, and 20‐cm AP thickness. Isocenter was set 25 cm away from the phantom longitudinal center to emulate the spatial distribution typically encountered in patients when the isocenter is located in the longitudinal axis coinciding with the umbilicus. Because of the phantom symmetry, we restricted the analysis to the anterior incidence. To protect seven29 detector electronics, the field size was reduced to 40 cm × 11 cm at isocenter (80 cm × 22 cm at detector distance, approximately). Field weights were optimized to have a uniform dose at the phantom mid‐coronal plane (Figure 3).

Once defined the treatment parameters, the plan was recalculated on a phantom of 30 cm length, 30 cm width, and 20 cm thickness. The phantom was set in seven different positions, shifted in the longitudinal direction so that the phantom center was successively at −60, −30, 0, 30, 60, 90, and 120 cm away from the isocenter. In this way, the complete length of the original phantom could be covered.

In the treatment room, seven29 detector was embedded in the RW3 phantom so that effective depth of measurement coincided with the mid‐coronal plane of a 20‐cm‐thick phantom. The treatment plan was delivered with the phantom successively located in the same seven positions described above so as to reproduce the geometry used in Eclipse. A gamma index analysis was performed with VeriSoft v5.1 software (PTW‐Freiburg, Freiburg, Germany) to compare calculated and measured dose distributions for each position.

#### 2.2.2 Axial planar analysis in the anthropomorphic phantom

**FIGURE 4:**
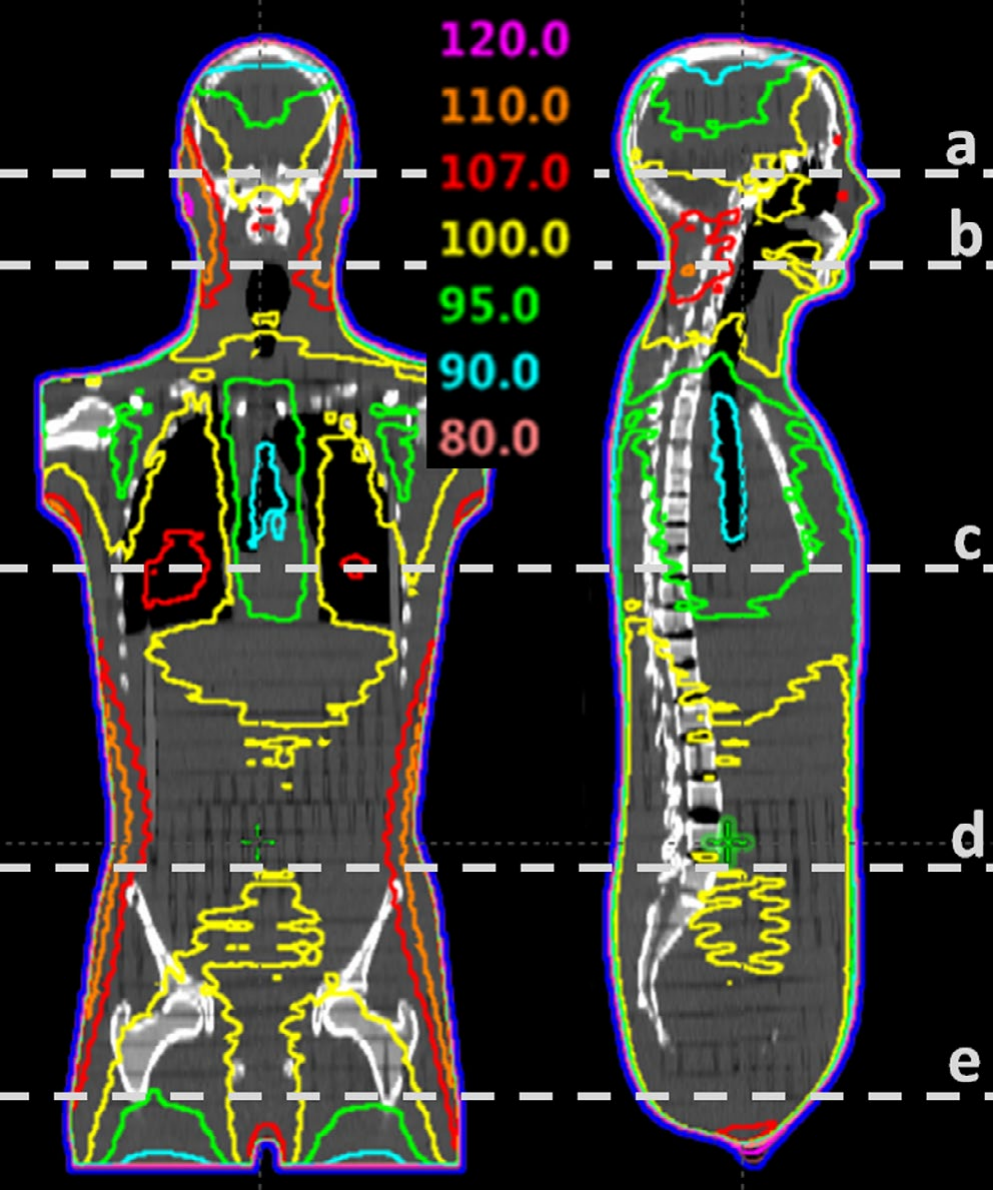
Isodose lines in the mid‐coronal and mid‐sagittal planes of the Alderson Radiation Therapy (ART) phantom for the institutional total body irradiation plan template. White dash lines show the axial planes measured with film

To validate the technique in a more realistic situation, we simulated the clinical conditions found in TBI treatments using an Alderson Radiation Therapy (ART) anthropomorphic phantom. The phantom, which represents the torso, neck, and head of a 73.5 kg human male, was CT scanned according to our clinical protocol for TBI patients. Correspondingly, treatment planning was carried out from our institutional plan template for TBI treatments (Figure 4). Since the phantom is transversally segmented into 1" (2.54 cm) sections, radiochromic films were trimmed to conform to the phantom's cross‐section and then inserted between the layers at five different heights in order to evaluate axial dose distributions (shown as white dash lines in Figure 4).

Once delivered the complete AP‐PA treatments (no lung shielding was employed), film dose conversion was performed by means of a home‐made implementation of the triple‐channel method,^18^, ^19^ which also incorporates the correction for lateral scanner artifacts and the use of a control film piece.^20^, ^21^ The comparison between measurement and calculation for each axial dose distribution was performed through a gamma index analysis performed with SNC Patient v6.7.2 software (Sun Nuclear, Florida, USA).

### 2.3 Beam characterization and dose calculation accuracy in the presence of lung shielding

#### 2.3.1 Linear attenuation coefficient of Cerrobend

We measured the linear attenuation coefficient of Cerrobend, the material chosen to manufacture the shielding blocks, with a detector array seven29 and RW3 phantom slabs. Five Cerrobend slabs were made, each one of size 12 cm ×12 cm ×1 cm. We used an SSD of 200 cm, an effective depth of measurements of 10 cm, and a 0 gantry beam of 5 cm ×5 cm field size at the isocenter (10 cm ×10 cm at the phantom surface). Cerrobend slabs were positioned on the phantom surface, stacked on top of each other so as to measure with shielding thickness of 1, 2, 3, 4, and 5 cm. Although Cerrobend attenuation was derived from the central chamber readings, the use of a detector array allowed us to verify the homogeneity of each slab, discarding the presence of imperfections in thickness, bubbles, etc.

#### 2.3.2 Dose profiles underneath shielding blocks

Analogously to what was performed in the case of unshielded fields, we analyzed Eclipse dose calculation accuracy in the presence of lung shielding by measuring with a detector array seven29 embedded in RW3 solid phantom in the same three configurations described in Figure 2. The phantom, emulating a 20‐cm‐thick patient, was positioned at an SSD of 202.5 cm. In addition, a Cerrobend slab of 1 cm ×12 cm ×12 cm was centered on the phantom surface. Coronal plane dose measurements were performed at depths of 2.5, 10, and 17.5 cm with a field size of 8 cm ×40 cm at isocenter. Calculation accuracy was evaluated through the comparison between measured and predicted longitudinal and transversal dose profiles.

#### 2.3.3 Depth–dose curve for shielded static and arc fields

From the previous measurements with the 2D detector array seven29, we analyzed depth–dose Eclipse accuracy in the presence of Cerrobend blocks in the three situations mentioned above (setup A, B, and C). To do this, we compared the dose given by the central chamber of the detector array (previously calibrated with an open field at 10 cm depth and 202.5 cm SSD) in every setup and depth of measurement with the corresponding dose provided by Eclipse.

#### 2.3.4 Block thickness determination and effective lung protection

Mean lung dose depends on the specific shape of the organ and, in particular, on the way this interacts with the large longitudinal penumbra due to shielding blocks. As a consequence, a simple estimation of the appropriate block thickness based on the Cerrobend linear attenuation coefficient could lead to a significant miscalculation. In order to perform a more realistic estimation of the mean lung dose in the presence of shielding blocks, we reproduced in Eclipse the shape and position of 1‐cm‐thick blocks in real patient CT scans in the same way they would be set during treatment. From Eclipse dose calculation, we then analyze the effect of lung shielding on dose distributions and lung dose–volume histograms and the adequacy of the block thickness used.

## 3 RESULTS

### 3.1 Arc‐based technique vs. MATBI and conventional AP/PA techniques

**FIGURE 5:**
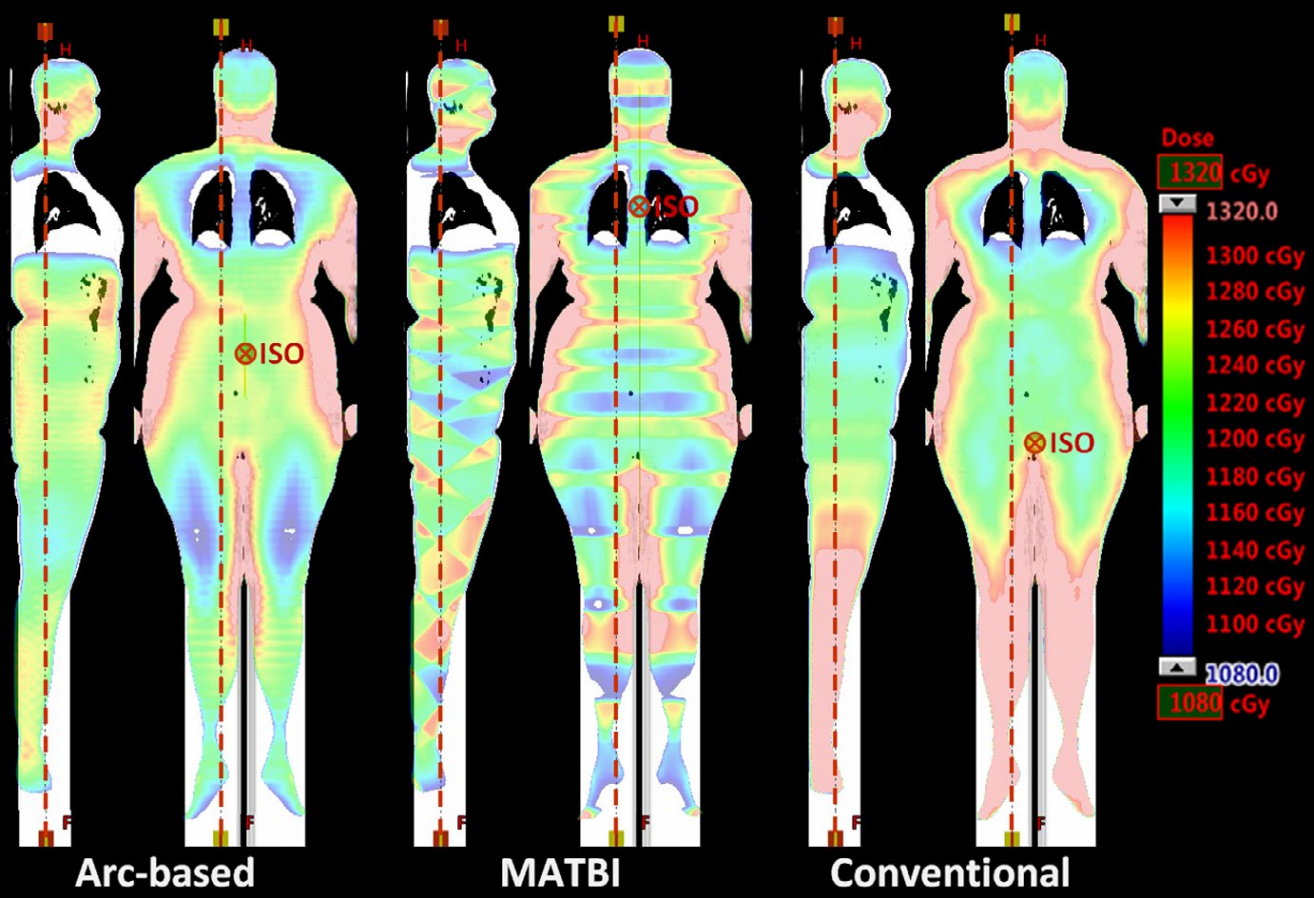
Calculated dose distribution for arc‐based, MATBI, and conventional extended source‐to‐surface distance techniques. Red dashed lines indicate the position of the corresponding coronal and sagittal planes. Isocenter position, which is different for each technique, is shown in every coronal plane. Rectangular regions in white in the inferior part of the images are due to the artificial expansion of the CT images necessary to have the full body in the same study

In Figure 5, it is shown the comparison between coronal and sagittal dose distribution for our technique and those obtained with MATBI and conventional AP/PA techniques. The prescription total dose was 1200 cGy, to be delivered in four daily fractions of 300 cGy each. For each technique, the plan was normalized so that the mean dose in the whole body coincides with the prescribed dose. Calculated doses are represented by a color wash with the lowest and highest dose shown being 1080 cGy (90% of the prescribed dose) and 1320 cGy (110% of the prescribed dose), respectively. Dose in regions with less than 1080 cGy is not shown, while those regions with more than 1320 cGy are depicted in pink. Planning time devoted to optimizing arc‐field weights in the arc‐based techniques was estimated to be 15–20 min. Regarding the MATBI technique, the planning time could increase to 45–60 min due to the necessity in Eclipse of manually adjusting the weight of the 44 fields (22 fields per side) included in the plan. In the case of using an automatic optimization of beam weights as in the original MATBI technique, inverse planning is expected to take less than 15 min.^8^

**FIGURE 6:**
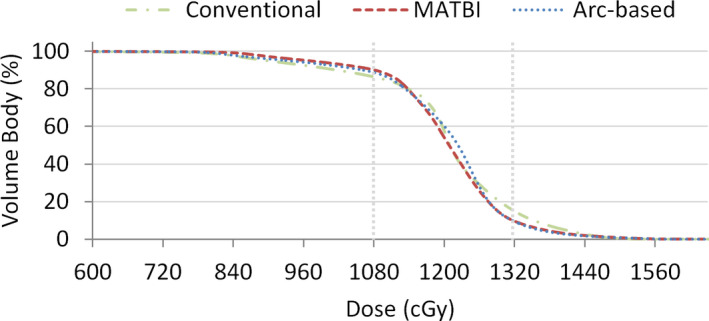
Comparison of the dose–volume histograms for the body for arc‐based, MATBI, and conventional extended source‐to‐surface distance techniques. Doses corresponding to ±10% of the prescription dose are shown in grey dotted lines

Figure 6 displays the dose–volume histogram (DVH) for the body corresponding to each technique. With respect to dose uniformity, it can be seen that no clinically relevant difference is found between arc‐based and MATBI technique. The volume of the body within ±10% of the prescription dose, V(±10), was 79.3%, 80.5%, and 71.4% for arc‐based, MATBI, and conventional techniques, respectively. The corresponding standard deviation of dose in the body for arc‐based, MATBI, and conventional techniques was 125, 118, and 140 cGy, respectively. Although the results presented in this work for the MATBI technique might be further improved by means of inverse‐planning, the value of V(±10) and the standard deviation of dose that we obtained for the MATBI plan are within the range of values published for this technique.^7^

**FIGURE 7:**
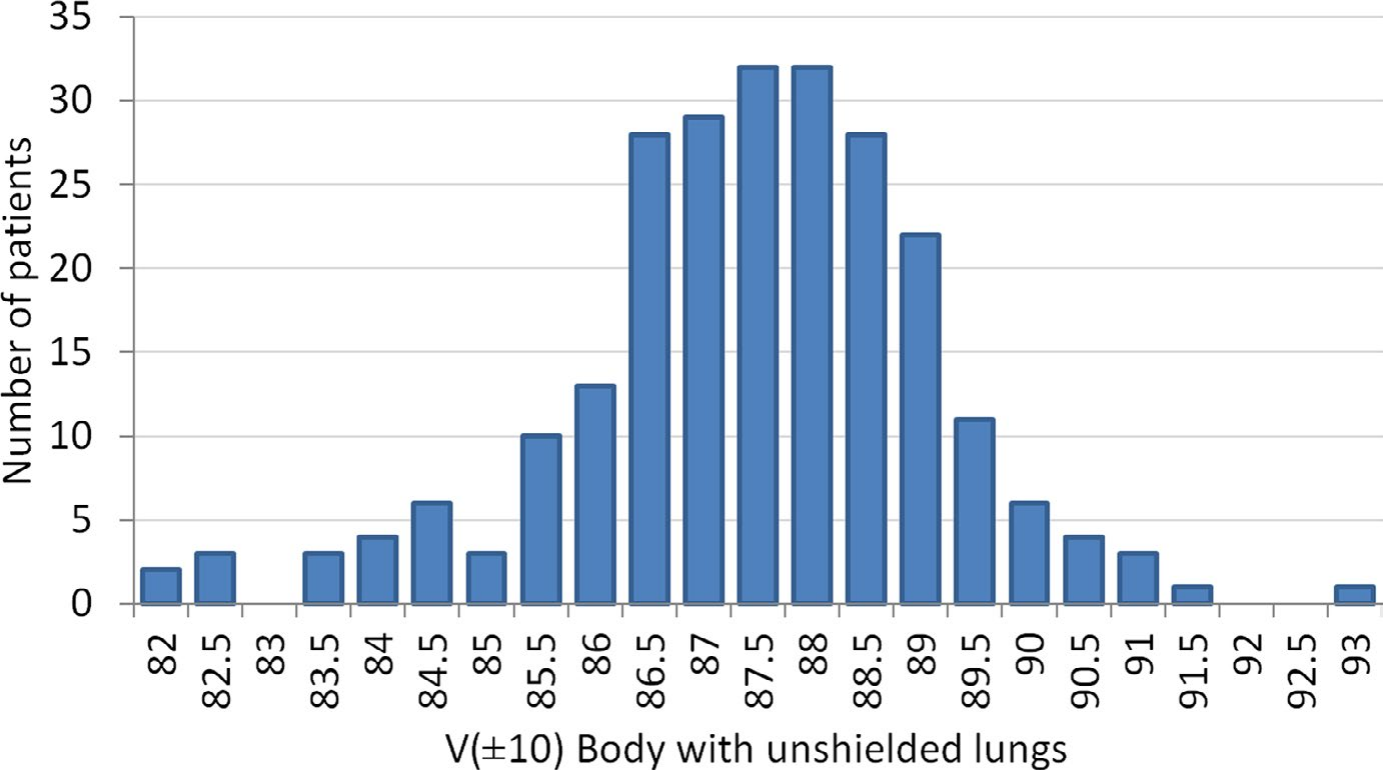
Histogram of the distribution of V(±10) values corresponding to the plan without shielding blocks for more than 240 patients treated with the arc‐based technique. Mean value of V(±10) is 87.2%, with a standard deviation of 1.7%

To have a better insight into the degree of uniformity that can be reached with the arc‐based technique, we present in Figure 7 the histogram of the distribution of V(±10) values for more than 240 patients treated with this technique. The mean value of the uniformity parameter V(±10) is 87.2%, with a standard deviation of 1.7%. Since lung shielding is not routinely considered during treatment planning in our institution, V(±10) values included in the histogram correspond to the unshielded lung plan. The relation between V(±10) values with and without shielding blocks is variable, depending mainly on patient and lung sizes. As an example, in the case of the patient selected for the current comparison of techniques, V(±10) for the plan with shielding blocks was 79.3%, while the corresponding value for the unshielded case was 86.0%.

**FIGURE 8:**
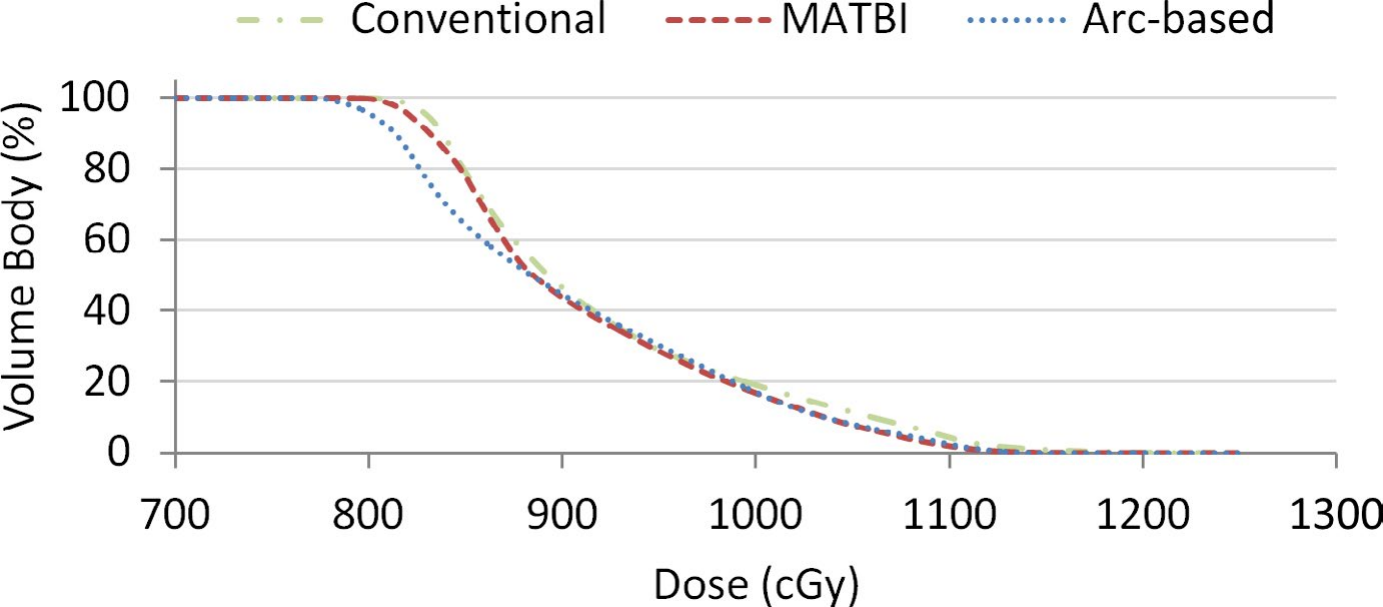
Comparison of the dose–volume histograms for the shielded lungs for arc‐based, MATBI, and conventional extended source‐to‐surface distance techniques. Following our clinical practice, a 1‐cm‐thick Cerrobend shielding was used for the three techniques

**FIGURE 9:**
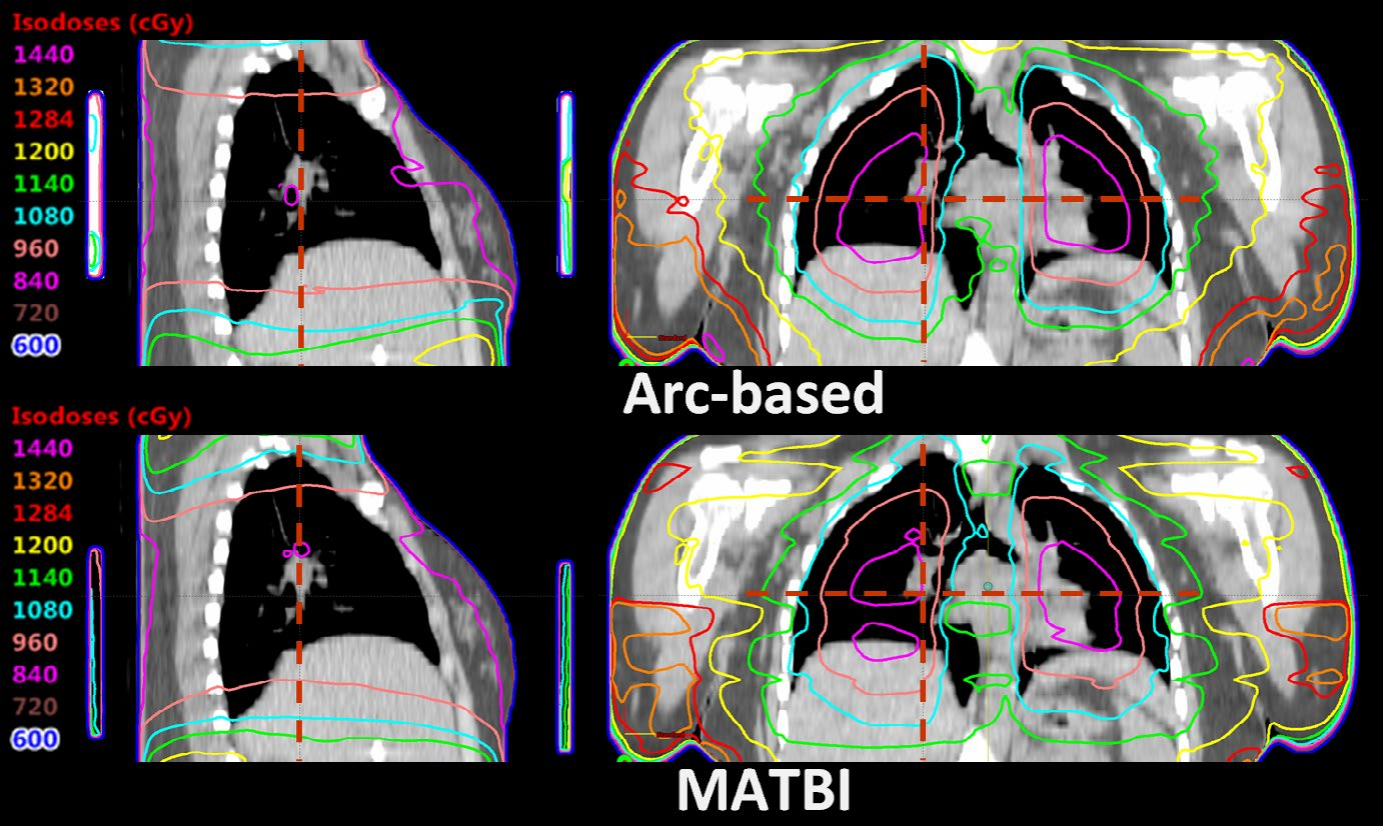
Calculated dose distribution for arc‐based and MATBI for the pulmonary region. The position of the 1‐cm‐thick Cerrobend lung shielding is shown in the sagittal planes

**FIGURE 10:**
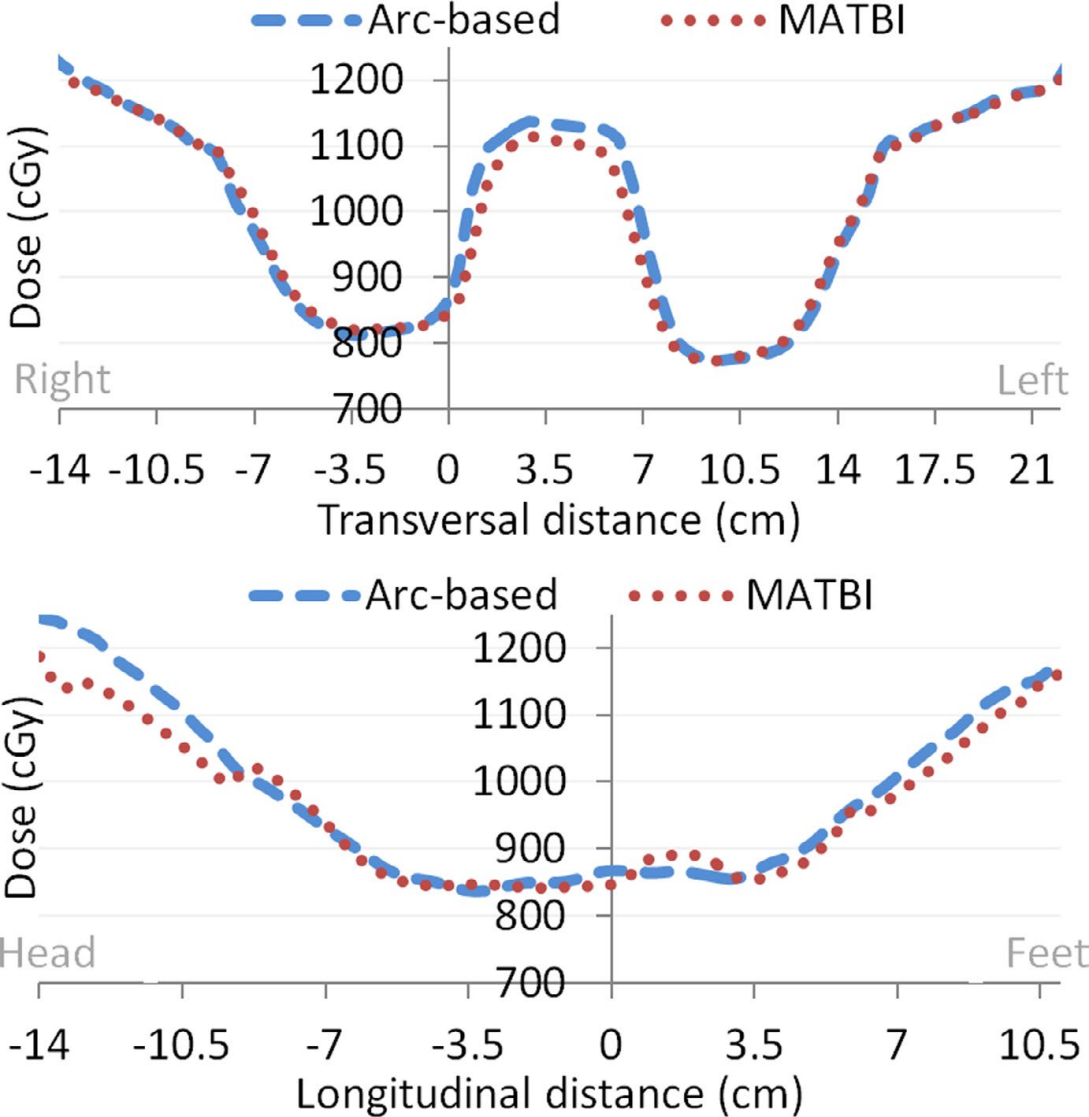
Comparison of transversal and longitudinal dose profiles for arc‐based and MATBI in shielded lungs. The profiles correspond to the red dotted lines shown in Figure 9

Concerning the dose delivered to shielded lungs by the three techniques being compared, the resulting dose–volume histograms are shown in Figure 8. Mean lung dose (±1standard deviation) corresponding to arc‐based, MATBI, and conventional techniques was 906 cGy (±87 cGy), 913 cGy (±78 cGy), and 921 cGy (±85 cGy), respectively. While in MATBI technique the isocenter longitudinal position is set centered in the lungs to have a narrower penumbra, in our technique, the isocenter is located near the umbilicus to reduce the number of arcs required to reach acceptable dose uniformity. Then, it is worth analyzing if there is a change in lung shield penumbra due to this isocenter displacement. Figure 9 displays the coronal and sagittal dose distributions restricted to the lung region for both arc‐based and MATBI techniques. Transversal and longitudinal dose profiles corresponding to the red dash lines in Figure 9 are given in Figure 10. From these figures, it can be seen that there is no appreciable variation in transversal or longitudinal shielding penumbra between arc‐based and MATBI techniques.

Another point of concern about our technique is the effective dose rate during treatment, that is, the average amount of radiation delivered per unit of time to the different organs at risk. Dose rate is believed to influence the biological effect of radiation, which in turn might affect normal tissue toxicity.^22^, ^23^, ^24^, ^25^ Some clinical protocols require low dose rate treatment at the rate of 5 to 10 cGy/min.^3^ Other studies, however, have suggested that a higher dose rate TBI can be given safely if the total doses are adequately fractionated.^26^, ^27^, ^28^, ^29^, ^30^, ^31^ For example, in the Ottawa Hospital Cancer Center, the dose rate for TBI has consistently been greater than 50 cGy/min, which is much higher than most published series. Yet the rates of clinically significant radiation pneumonitis are below 20% and fatal radiation pneumonitis <5%.^30^ We estimate the effective dose rate at a certain point through the quotient between the dose per fraction in that point given by Eclipse and the beam‐on time during which that point in under direct radiation beam. Four points of interest were considered for this evaluation, centered on the following organs at risk:

- P1: thyroid gland,
- P2: left lung,
- P3: left kidney,
- P4: superior part of the intestines,
- P5: inferior part of the intestines.

Dose at points P1, P2, and P3 are expected to be related to hypothyroidism, pneumonopathy, and kidney dysfunction, which are three of the most important long‐term sequelae.^1^ Regarding points P4 and P5, they are associated with gastrointestinal acute toxicity, one of the most common early side effect.^32^ The effective dose rate for points P1 to P5 were, respectively, 18.0 cGy/min, 11.5 cGy/min, 18.5 cGy/min, 17.4 cGy and 23.6 cGy/min for the arc‐based technique and 12.5 cGy/min, 8.0 cGy/min, 11.7 cGy/min, 11.2 cGy and 14.5 cGy/min for the MATBI technique. The higher effective dose rate in the arc‐based technique is the consequence of using a repetition rate of 80 MU/min for lungs and 400 MU/min for the rest, instead of 50 and 300 MU/min as in the original MATBI technique. Despite the increment in the calculated effective dose rate, the values we obtained for the arc‐based technique are still lower than those published in the aforementioned literature.^30^, ^31^ It is worth mentioning that no shielding or compensator other than lung shielding was employed to reduce dose or dose rate in critical organs at risk, in agreement with the findings in a recent survey.^31^

Finally, we compare arc‐based and MATBI techniques in terms of treatment delivery time. Arc‐based technique requires 32 min to deliver 4904 MU corresponding to both AP and PA treatments, with a repetition rate of 80 MU/min for those fields irradiating lungs and 400 MU/min for the rest. Moreover, in the case of the MATBI technique, the treatment time is 52 min for a total of 5239 MU and repetition rate of 50 MU/min for lungs and 300 MU/min for the rest of the fields.

### 3.2 Beam characterization and dose calculation accuracy at an extended distance

#### 3.2.1 Depth–dose curve, floor backscatter, and skin dose ‐ static fields

**FIGURE 11:**
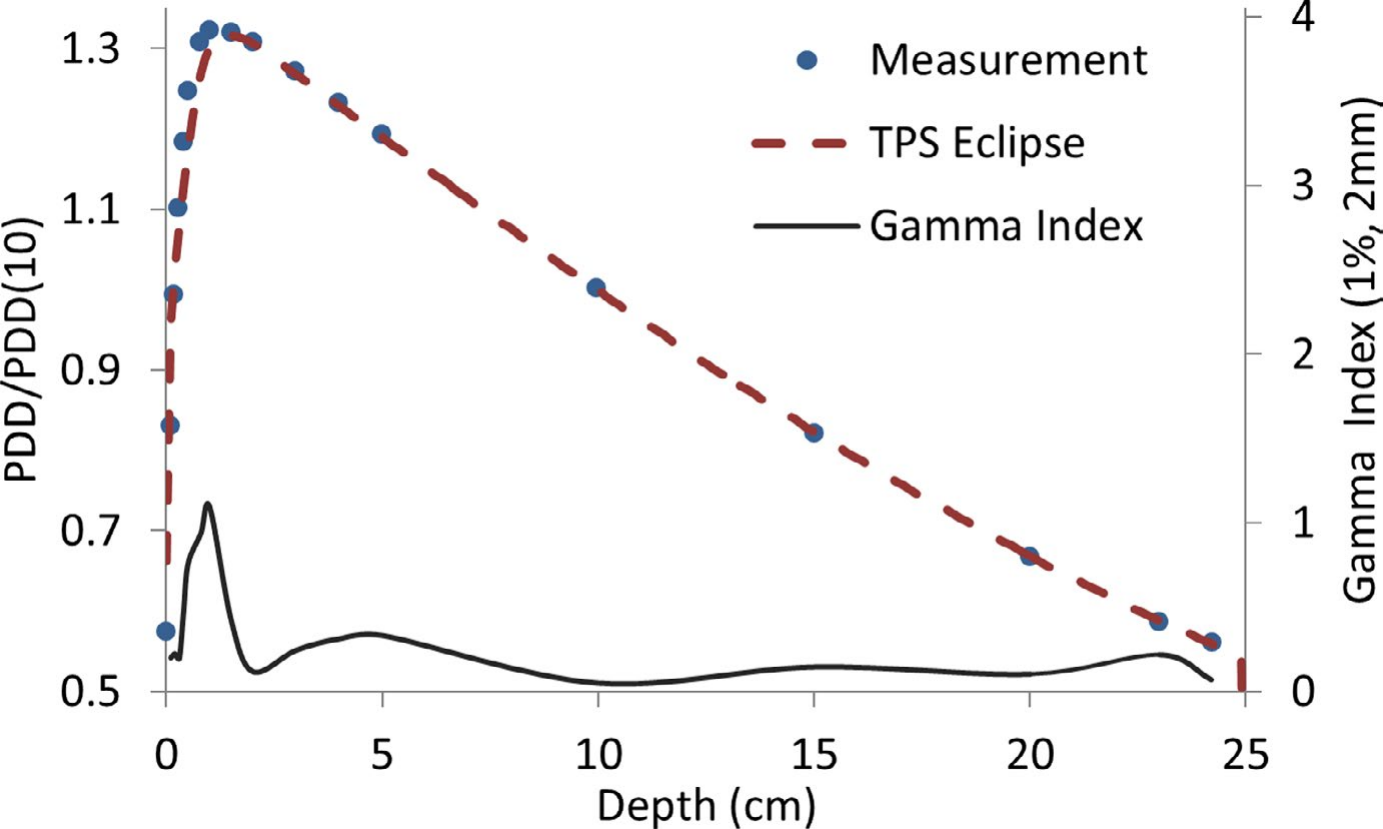
Measured and calculated percentage depth dose curves (normalized at a depth of 10 cm), along with the results of the one‐dimensional gamma index analysis for 1%/2 mm passing criteria

Figure 11 displays measured and calculated PDD curves, both normalized to a depth of 10 cm. It is also shown in this figure the results of the gamma index analysis for 1%/2 mm passing criteria. As can be seen, there is a high agreement between both curves, even in the build‐up region. Interestingly, correspondence between curves in the deepest region of the phantom could be considered as an indication of the negligible value of the floor backscatter contribution.

**FIGURE 12:**
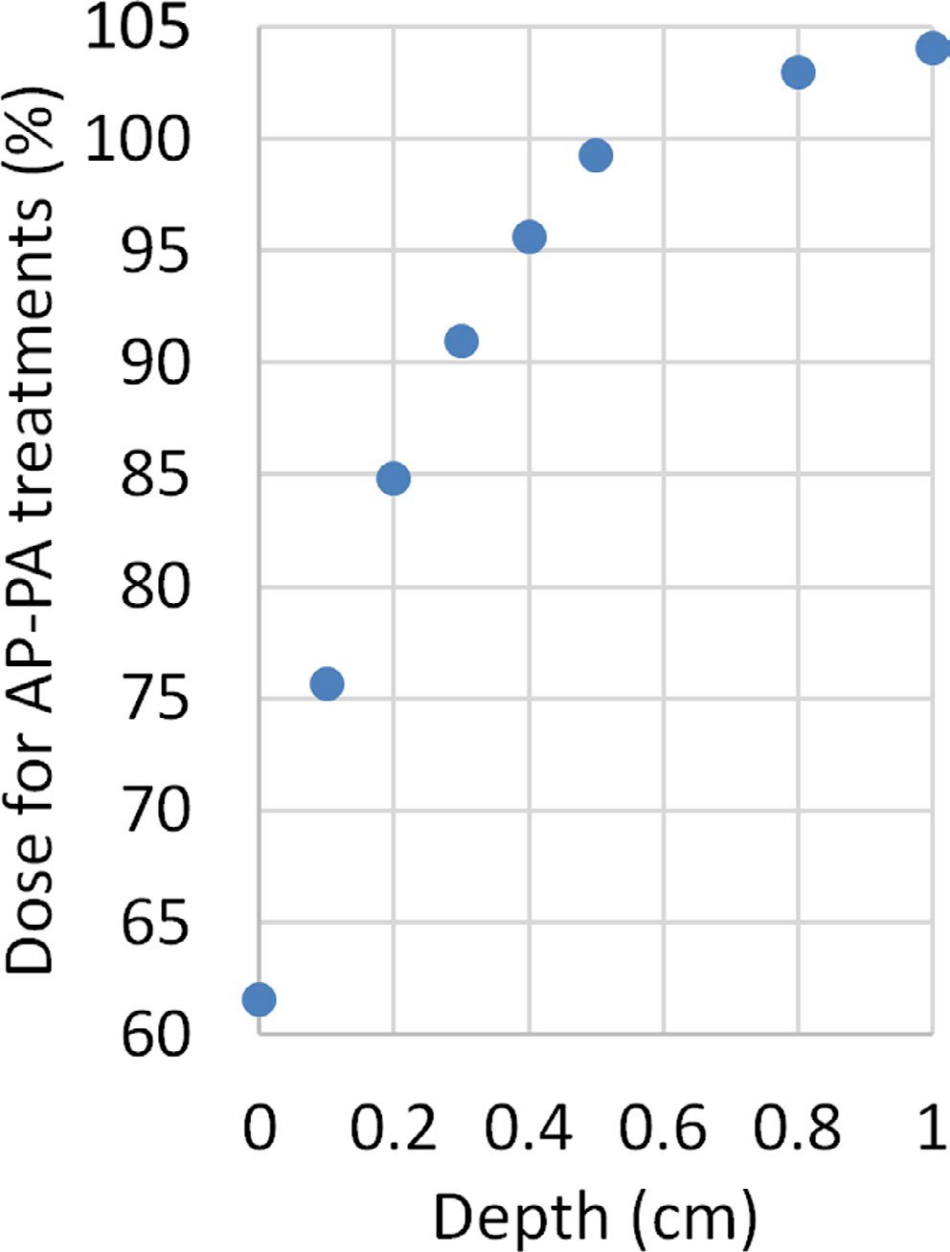
Depth–dose curve (normalized at the mid‐coronal point) corresponding to the full AP‐PA treatment, for the depth range [0 1] cm. percentage depth dose at 2‐mm depth reaches a value of 85%

From the addition of both entrance and exit dose contributions, we can estimate the value of the skin dose for the full AP‐PA treatments. Figure 12 shows this depth‐dose curve for the first centimeter. In this figure, dose values are normalized to the dose at the mid‐coronal point (usually, the prescription point). It can be seen that the percentage dose becomes higher than 85% once a depth of 2 mm is reached. Based on these results and our previous clinical experience with lateral‐opposed technique at a large distance, it was decided not to use beam spoiler to increase skin dose.

#### 3.2.2 Beam quality index and reference absolute output

Beam quality index TPR_20,10_, measured at an extended distance, has a value of 0.671. The value obtained from Eclipse is 0.663 (relative difference of −1.2%), while the beam quality index corresponding to a standard distance is 0.669.

Absolute absorbed dose for TBI conditions has a measured value (corrected for daily output) of 0.206 cGy/MU and a calculated value of 0.208 cGy/MU, which represents a relative difference of 1.0%.

#### 3.2.3 Beam profile, penumbra, and out‐of‐field dose

**FIGURE 13:**
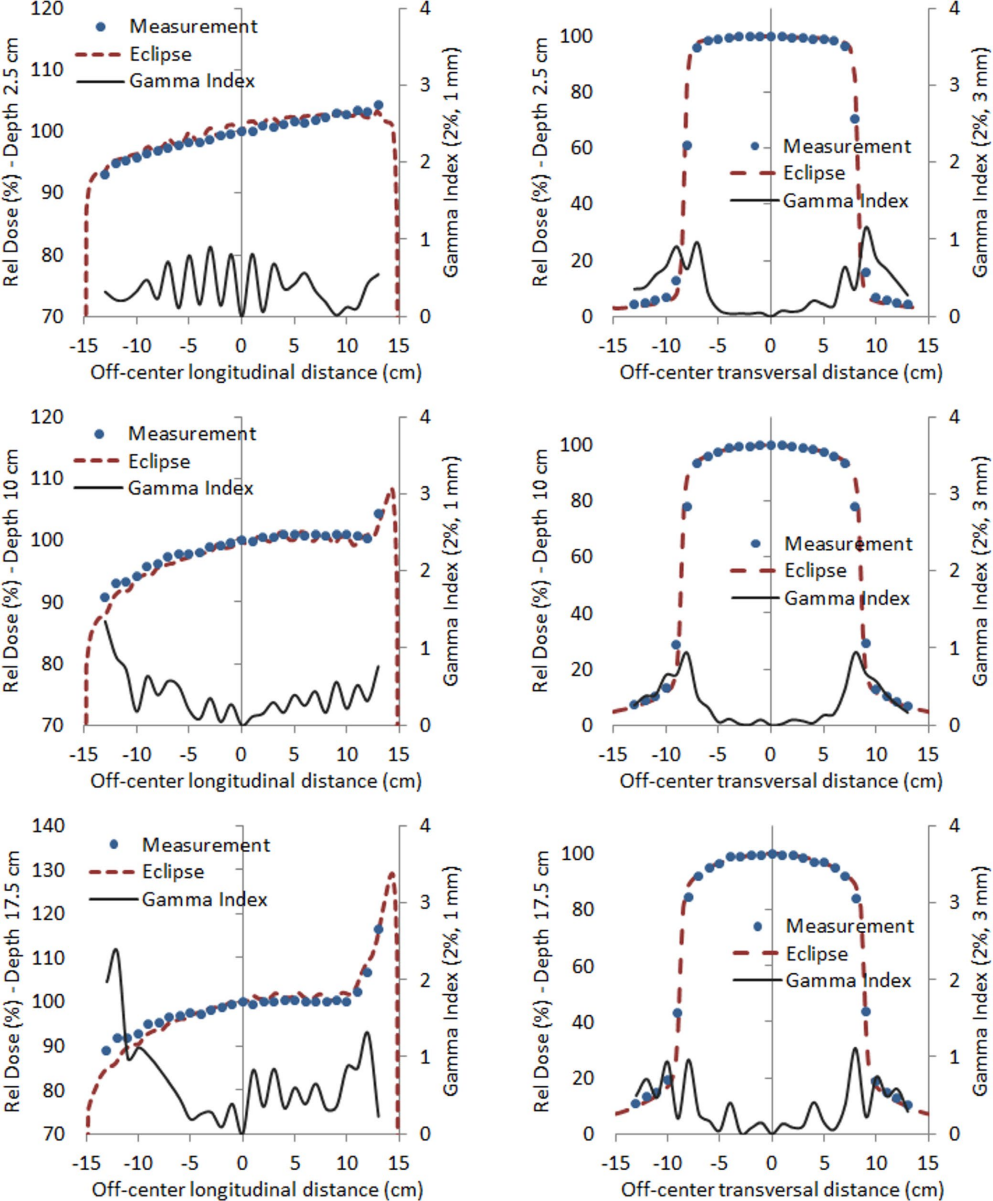
Measured and calculated dose for setup C and the respective one‐dimensional gamma index analysis for longitudinal (1st column) and transversal (2nd column) profiles, corresponding to a depth of 2.5 cm (1st row), 10 cm (2nd row), and 17.5 cm (3rd row)

Coronal dose distributions were obtained at depths of 2.5, 10, and 17.5 cm with a detector array seven29 in the three conditions depicted in Figure 2a–c. The reduced field size used, albeit different from the treatment field size, gives us the possibility to evaluate Eclipse accuracy in open‐field, penumbra, and out‐of‐field regions. The comparison was carried out through a one‐dimensional gamma index analysis on the longitudinal and transversal profiles crossing the central detector. We employed a passing criterion of 2%/3 mm for transversal profiles and a more restrictive criterion of 2%/1 mm for longitudinal profiles, where only the open‐field region is present. Figure 13 shows the profiles and gamma index results for the case where the phantom is positioned 30 cm away from the 0 gantry beam axis in the longitudinal direction (setup C). Similar results were obtained in the other two measurement conditions (setup A and B). A very good agreement between curves can be seen, even in the out‐of‐field region. Discrepancies in the penumbra region, although acceptable in magnitude, are expected due to the ionization chamber volume effect and low spatial resolution of the detector array. It is also noticeable in the longitudinal profiles the effect of the angular discretization made by Eclipse.

#### 3.2.4 Depth–dose curve for arc fields

**FIGURE 14:**
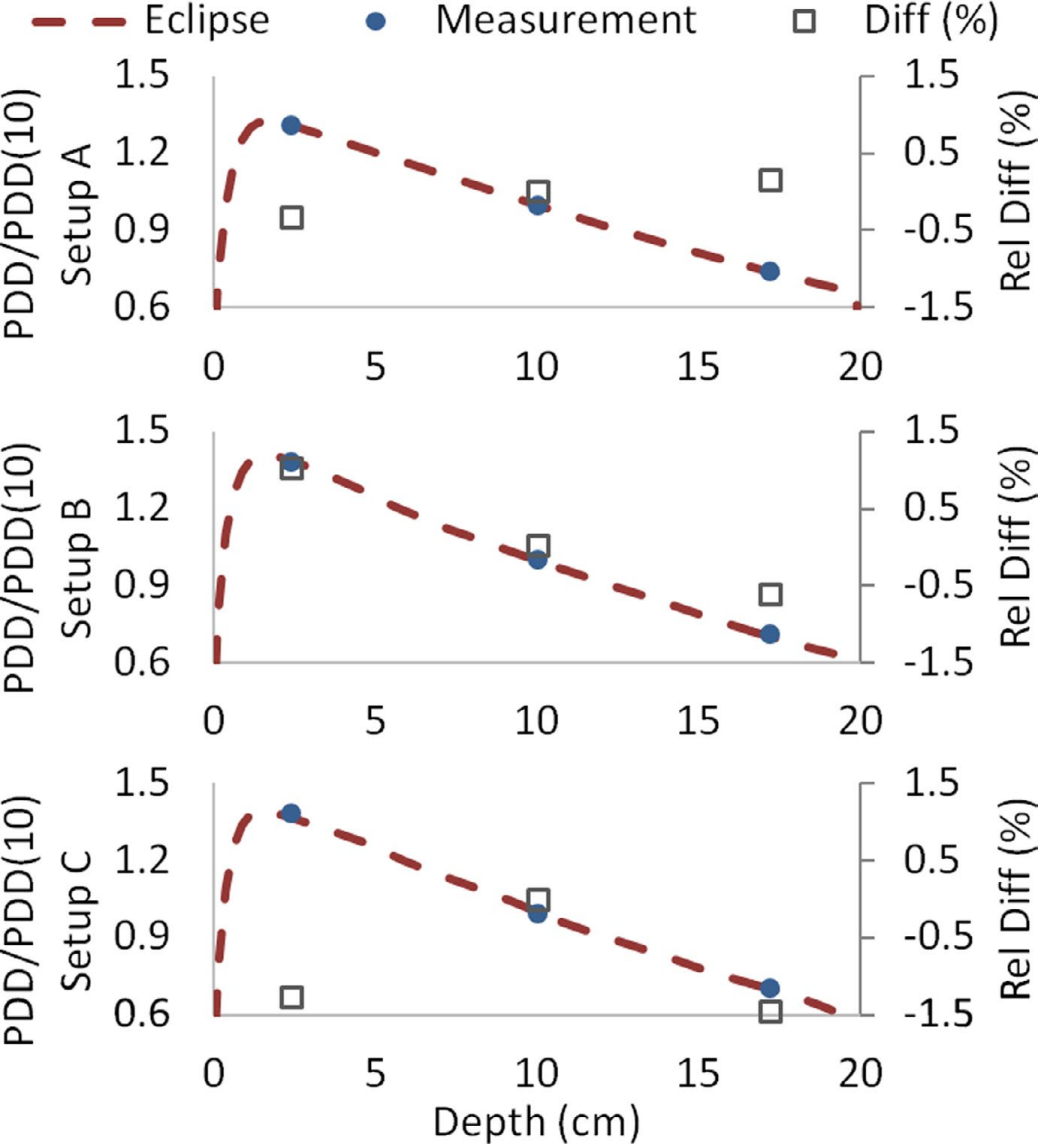
Calculated percentage depth dose curves and the measured values at 2.5, 10, and 17.5 cm for setup A, B, and C. Relative differences in dose are always below 1.5%

Eclipse depth–dose calculation accuracy for arc fields was evaluated by the central chamber's readings of the detector array from the previous measurements at depths of 2.5, 10, and 17.5 cm. Figure 14 shows the PDD/PDD(10) calculated curve for the three setups used, along with the measured values and the corresponding relative differences. It can be seen that relative differences in dose are always below 1.5%. Moreover, percentage differences between calculated and measured absolute dose at 10‐cm depth for each setup were 0% for setup A (detector array calibrated in this setup), −1.5% for setup B, and −0.7% for setup C.

### 3.3 Evaluation of dose calculation for the complete treatment plan

#### 3.3.1 Coronal planar analysis in solid phantom

**FIGURE 15:**
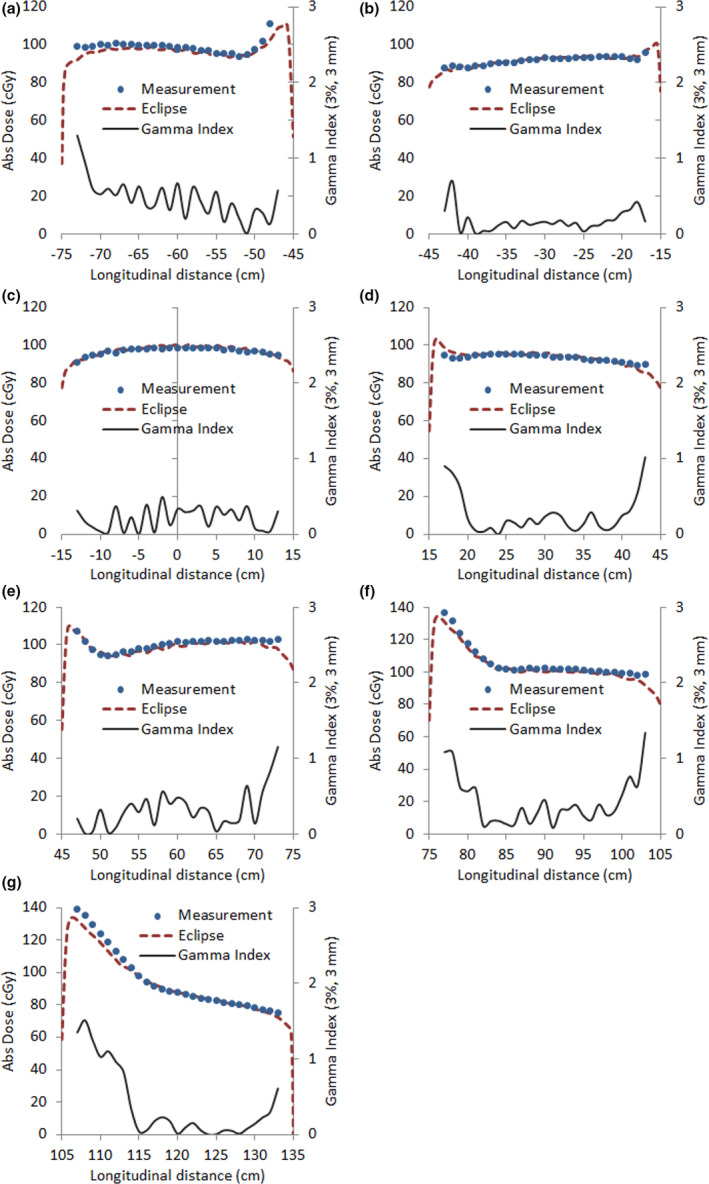
Measured and calculated longitudinal dose profiles (in cGy) and the corresponding gamma index analysis for seven detector positions. Longitudinal detector displacements were: (a) −60 cm, (b) −30 cm, (c) 0 cm, (d) 30 cm, (e) 60 cm, (f) 90 cm, and (g) 120 cm

In order to evaluate Eclipse calculation performance for a typical treatment plan, measurements were carried out with a seven29 detector embedded in the RW3 phantom so that effective depth of measurement coincided with the mid‐coronal plane of a 20‐cm‐thick phantom. To cover the entire length of interest, the phantom center was successively positioned at −60, −30, 0, 30, 60, 90, and 120 cm away from the isocenter. Two‐dimensional gamma index analysis with 3%/3 mm passing criteria gives an average value of 90.9% of points with γ<1 for the seven positions (range: 84.8%–93.8%), being most of the failing points in the lateral penumbra region or close to the longitudinal border of the phantom. Figure 15a–g shows both measured and calculated absolute dose along with the corresponding gamma index results, where the values displayed are restricted to the longitudinal profile for the sake of clarity. Points outside tolerance in the region of lateral penumbra are expected, in part, due to the volume averaging effect resulting from the finite size of the detectors (this can also be seen in transversal profiles in Figure 13). Irrespective of the reason for the disagreement, inaccuracies in the modeling of the penumbra are likely to be of no clinical relevance since lateral penumbra regions are always outside the patient. With respect to the regions near the longitudinal edge of the phantom, failing points are most probably the consequence of oblique incidence and a lack of appropriate scatter. As a consequence, gamma analysis results would probably improve if additional phantom materials were added in the longitudinal direction on both sides of the detector to create more convenient scatter conditions.

#### 3.3.2 Axial planar analysis in the anthropomorphic phantom

**FIGURE 16:**
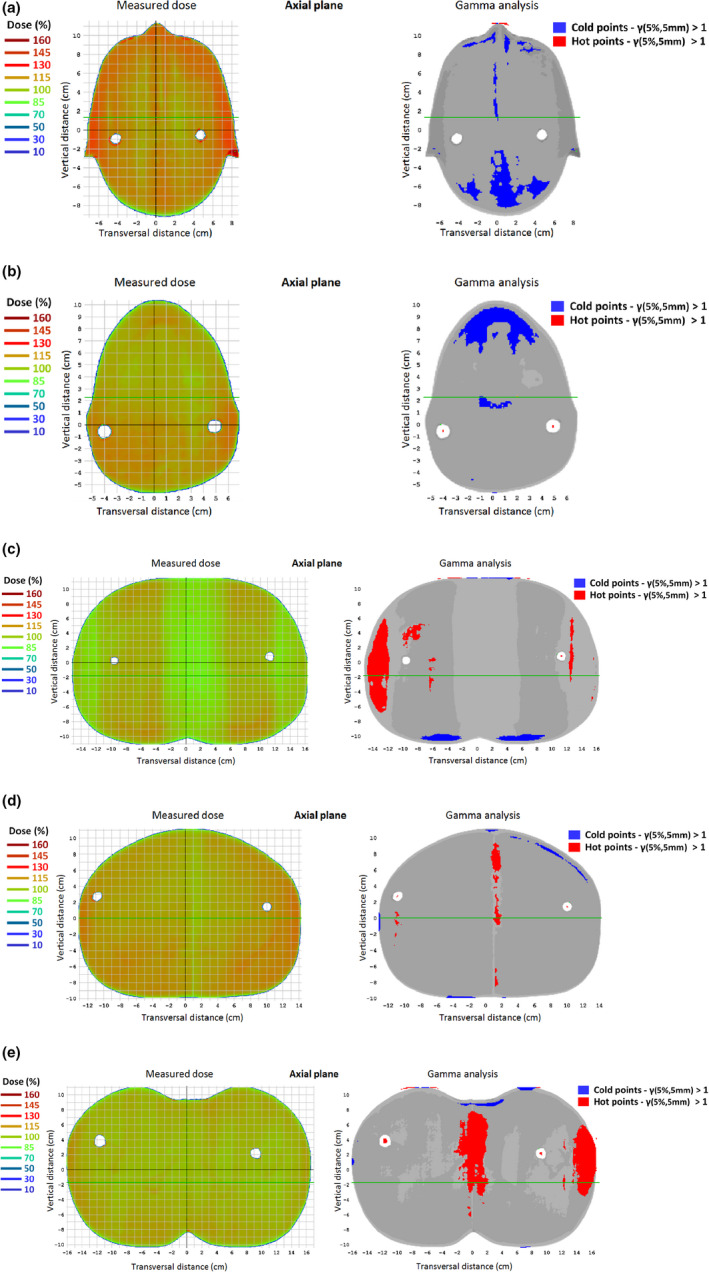
Measured dose plane (1st column) and the corresponding gamma index analysis (2nd column) with criteria 5%/5 mm for the five selected axial planes shown in Figure 4

Figure 16 presents both the measured dose plane and the corresponding gamma index analysis for each one of the five selected heights from the anthropomorphic ART phantom. Planar gamma index analysis with 5%/5 mm passing criteria results in an average value of 93.3% of points with γ < 1 for the five planes (range: 90.9%–98.1%). In this case, a less restrictive 5%/5 mm passing criteria was chosen to contemplate higher uncertainties arising in film dosimetry and phantom positioning.

### 3.4 Beam characterization and dose calculation accuracy in the presence of lung shielding

#### 3.4.1 Linear attenuation coefficient of Cerrobend

**FIGURE 17:**
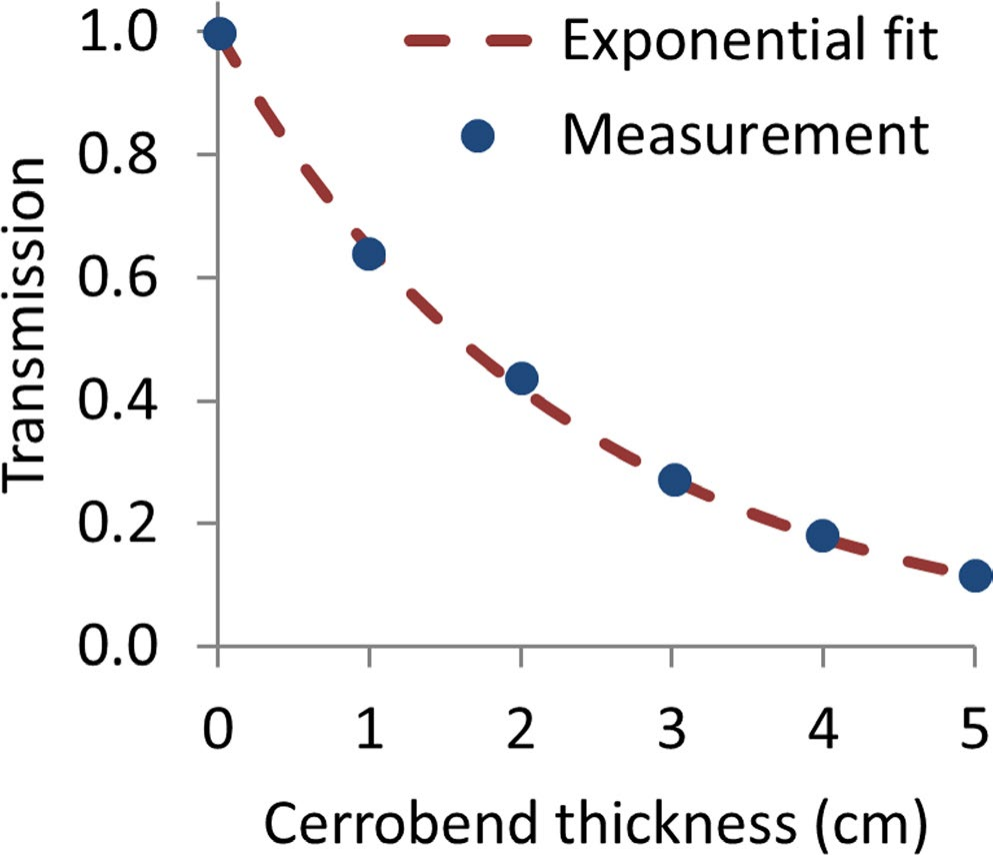
Transmission measured values as a function of Cerrobend thickness and the corresponding exponential curve fitting

Transmission values as a function of Cerrobend thickness are given in Figure 17, along with the corresponding exponential curve fitting. The linear attenuation coefficient for Cerrobend in TBI conditions, derived from the previous exponential fitting, has the value µ_TBI_ = 0.4339 cm^−1^. Based on this result, the use of 1‐cm‐thick shielding would yield a 35% attenuation of the incident intensity beam.

#### 3.4.2 Dose profiles underneath shielding blocks

In order to evaluate Eclipse dose calculation accuracy in the presence of lung shielding, the first step was to find the most suitable electron density value to be assigned to Cerrobend in Eclipse. To do this, a virtual Cerrobend slab of 1 cm × 12 cm × 12 cm centered on the phantom surface was created in Eclipse in a configuration emulating the one used during the measurement of coefficient µ_TBI_. Then, the Cerrobend electron density was modified following a trial and error method until the calculated beam attenuation coincided with the measured one. Thus, the resulting value for Cerrobend electron density (relative to water) was 10.55.

**FIGURE 18:**
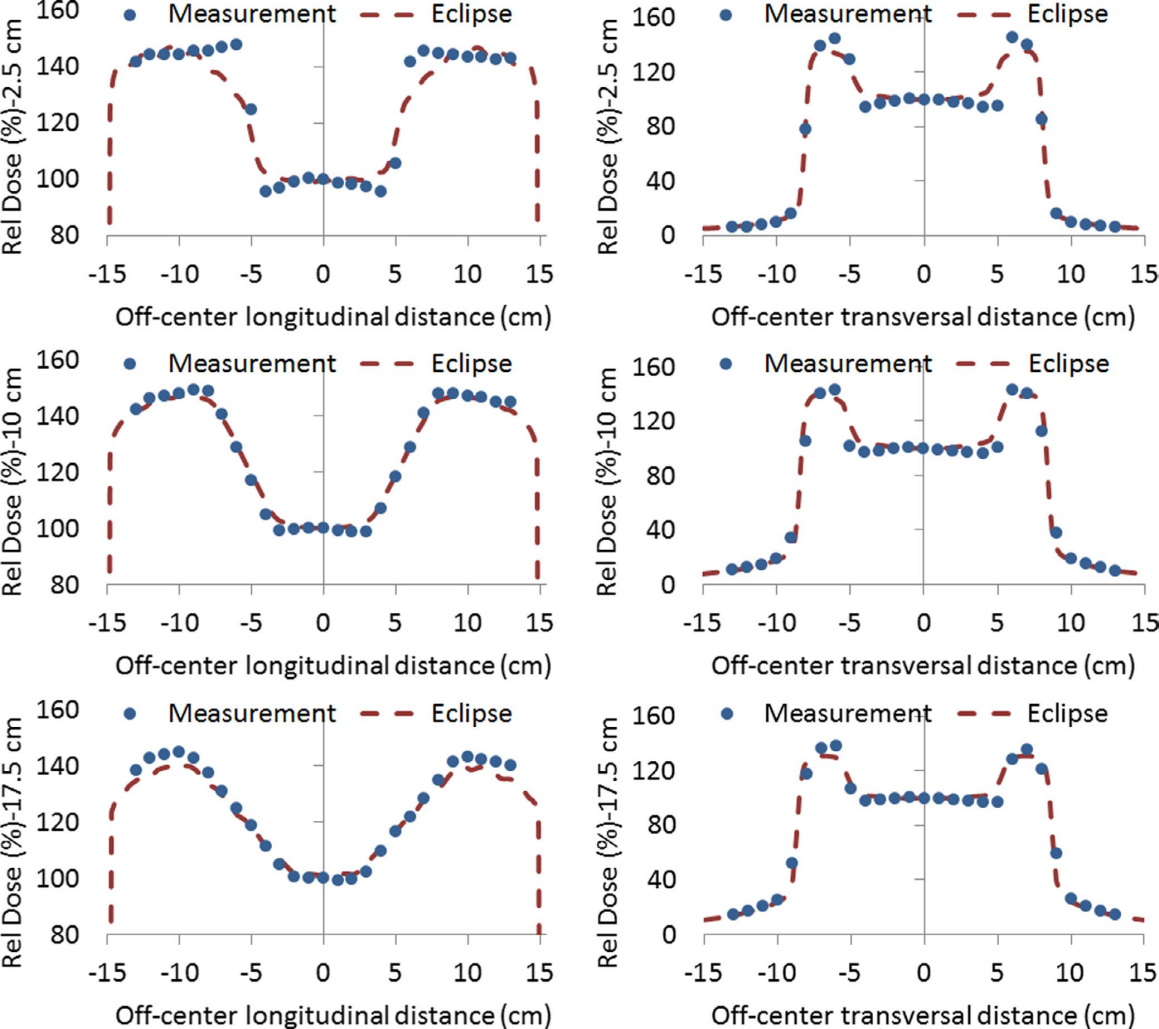
Measured and calculated dose for setup B in the presence of lung shielding for longitudinal (1st column) and transversal (2nd column) profiles, corresponding to a depth of 2.5 cm (1st row), 10 cm (2nd row), and 17.5 cm (3rd row)

Once determined the Cerrobend electron density value to be used in Eclipse, the three measurement configurations (setup A, B, and C) were reproduced in Eclipse. Figure 18 shows the comparison between measured and calculated doses (normalized to 0 gantry axis) for both transversal and longitudinal profiles obtained in setup B. The corresponding results for setup A and C exhibit a similar behavior. It can be seen from the first column in Figure 18 that the penumbra produced by the shielding slab in the longitudinal direction (i.e., in the direction perpendicular to the gantry rotation axis) is considerably wider than in the transversal case (second column in Figure 18), as a consequence of gantry rotation. This effect is accurately modeled by Eclipse, despite some discrepancy at 2.5 cm depth. With respect to transversal profiles, Eclipse slightly overestimates the width of the shield penumbra, with the disagreement being also in this case more noticeable at shallower depths.

#### 3.4.3 Depth–dose curve for shielded static and arc fields

**FIGURE 19:**
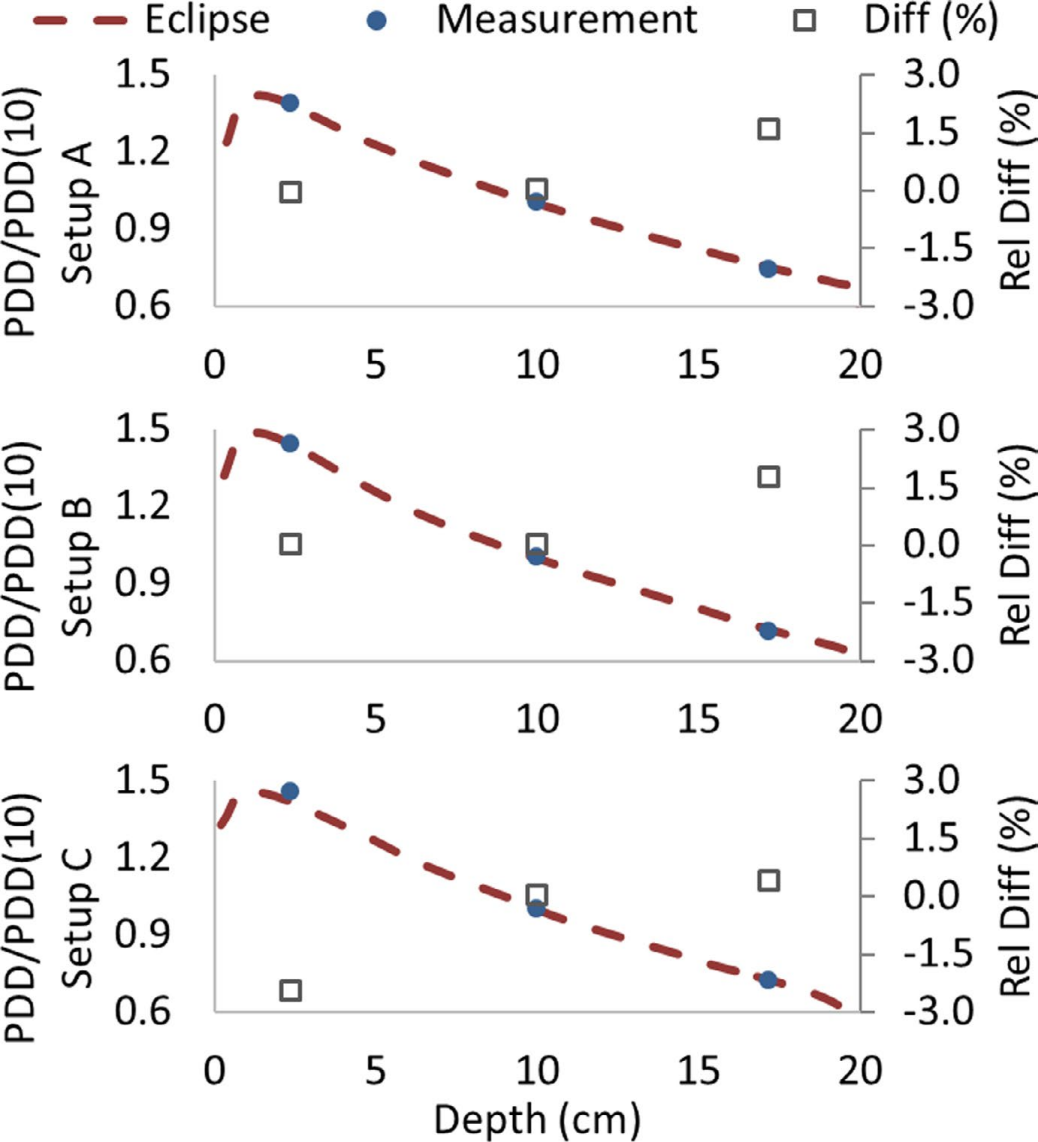
Calculated percentage depth dose (PDD) curves and the measured values at 2.5, 10, and 17.5 cm for setup A, B, and C in the presence of lung shielding

Similar to the open‐field case, we evaluated Eclipse depth–dose calculation accuracy for static and arc fields in the presence of lung shielding by the readings of the detector array central chamber at depths of 2.5, 10, and 17.5 cm. Figure 19 shows the PDD/PDD(10) calculated curve for the three setups used (see Figure 2), along with the measured values and the corresponding relative differences. Maximum differences in PDD/PDD(10) were 1.6%, 1.7% and −2.4% for setup A, B, and C, respectively. Concerning the absolute dose at 10‐cm depth, differences between calculated and measured values were −0.3%, −1.4%, and 0.6% for setup A, B, and C, respectively.

#### 3.4.4 Block thickness determination and effective lung protection

**FIGURE 20:**
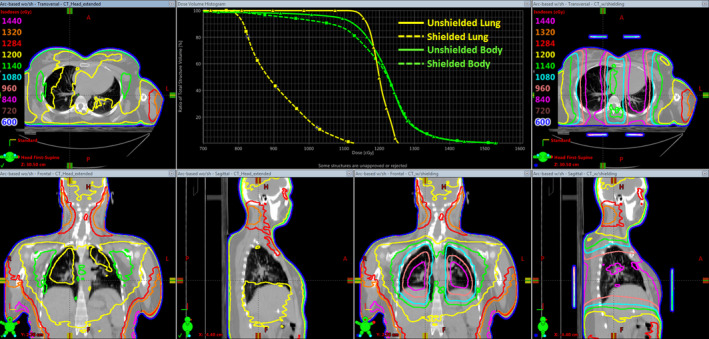
Volumetric dose distributions and lungs dose–volume histograms for a typical patient with a prescribed dose of 12 Gy. Orthogonal views on the right correspond to the shielded lung case (dashed dose–volume histogram (DVH) lines), while those on the left to the unshielded situation (solid DVH lines)

Having verified the accuracy of Eclipse dose calculation in the presence of lung‐shielding blocks at all depths in both the direct shielded and the penumbra regions, we made use of Eclipse to estimate the effective attenuation due to lung shielding. By CT scans from real TBI patients, we reproduced in the treatment planning system the position, size, and thickness of shielding as it was utilized during treatment. Relative electron density found previously was assigned to shielding structures and tridimensional dose calculation was redone in order to compare lung dose with and without shielding. Figure 20 shows three‐dimensional (3D) dose distribution comparison and lungs dose–volume histogram for a typical patient with a prescribed dose of 12 Gy. As a result of the previous analysis, we found that 1‐cm‐thick lung shielding leads to an average attenuation in a mean lung dose of 21.4%. This effective attenuation is considered adequate to maintain the mean lung dose below the recommended value of 10 Gy^22^, ^23^ in all our treatment schemes.

## 4 DISCUSSION

Total body irradiation has been used for over decades, being bilateral or AP/PA two‐dimensional TBI at an extended distance the most commonly used technique. Literature suggests that measured and expected doses should agree to within ±5% and dose uniformity on the patient should be within ±10% of the prescribed dose.^1^, ^3^ In order to achieve such uniformity, it is necessary to know patient thickness and contour for different sections of the body. Over the recent years, several publications have studied 3D‐based and volumetric modulated arc approaches to improve dose homogeneity with a wide range of complexity.

In our institution, we have commissioned a straightforward TBI treatments technique based on standard treatments 6 MV x rays, without spoiler, modeled in Varian Eclipse v13.6 treatment planning system. This allows us to perform patient‐specific 3D treatment plan, based on full‐body CT scan, with an entire contouring and planning time of 60 ± 30 min (considering parallel dose calculation over 12 CPU processors).

Treatments are delivered in two machines, a Varian Trilogy and a Varian 2100CD. Despite not being purposely matched to be dosimetrically equivalent, both machine models agree within 1% in output factors and within 1%/1mm in profile and PDD curves. This allows us to fulfill the requirement of a back‐up machine that guarantees the completion of treatments in case of machine breakdown.

Beam characterization and absolute dosimetry were performed at extended treatment distance (~200 cm), resulting in a high correspondence between modeled and measured depth–dose profiles for both static and arc conditions. Planar dose assessment was carried out in order to evaluate transversal and longitudinal profiles, with a very good agreement between curves, even in the out‐of‐field region. Acceptable discrepancies were observed in the penumbra region due to the ionization chamber volume effect, detector spatial resolution and, in longitudinal profiles, due to angular discretization made by Eclipse. It is worth noting that the entire validation was performed with an algorithm Eclipse AAA commissioned at standard SSD, confirming that no new machine modeling is required with this algorithm at an extended SDD of approximately 200 cm.

The complete treatment plan was evaluated by means of RW3 solid phantom and ART anthropomorphic phantom, with both 2D ion chamber array detector seven29 and EBT3 Gafchromic films.

Lung shielding is achieved in our institution by patient custom‐made 1‐cm‐thick Cerrobend shielding blocks derived from CT scan images. The high accuracy of dose calculation carried out by Eclipse in the presence of lung shielding provided us with a more reliable estimation of mean lung dose than that obtained from point dose attenuation measurements. Thus, lung shielding was found to lead to an average attenuation in mean lung dose of approximately 20% (significantly lower than the 35% value derived from the attenuation curve). Based on these results, 1‐cm‐thick shielding was deemed to be sufficient to keep total the mean lung dose below the recommended value of 10 Gy in all treatment schemes.

## 5 CONCLUSIONS

We successfully implemented in our institution a TBI treatment procedure based on a patient's specific full‐body CT image and volumetric treatment planning in a commercial treatment planning system (Varian Eclipse v13.6) commissioned for standard SSD treatments.

The proposed technique allows us to optimize the actual dose distribution, including regions with large heterogeneities such as thorax, and to verify the achieved dose uniformity in the entire volume of the body. The reduced number of fields we employed makes it possible to reach a satisfactory dose uniformity through manual optimization in a straightforward process. The technique has also proved to be robust enough to be applied in a wide range of patient size, from pediatric to adult overweight patients.

Another important characteristic of this technique is that, since the patient is positioned on a couch on the floor underneath the gantry, it imposes no requirement on the treatment room size. This attribute gave us the possibility of commissioning the technique in different machines, this becoming essential to fulfill the requirement of having a back‐up machine. It is also worth noting that no ancillary equipment is needed, except for a simple wooden‐made couch, making this technique robust enough to be easily implemented in other clinics.

At present, we had successfully treated over 240 patients with this technique. In vivo diode dosimetry verified an absolute mean dose agreement between planned and delivered dose better than 5% (tolerance value) in 93% of patients, being all patient's in vivo dosimetry inside 10% (action level value) dose agreement.

## CONFLICT OF INTEREST

The authors have no conflict of interest to disclose.

## AUTHOR CONTRIBUTION

León G. Aldrovandi: technique conception, study design, data acquisition, data analysis, manuscript writing, and revision; Rubén O. Farías: study design, data acquisition, data analysis quality control, manuscript writing, and revision; María F. Mauri: study discussion, data analysis review, manuscript critical review; María L. Mairal: manuscript critical review and supervision. All authors have read and approved the final manuscript.

## Data Availability

The data that support the findings of this study are available from the corresponding author upon reasonable request.
